# Are depression and poor sexual health neglected comorbidities? Evidence from a population sample

**DOI:** 10.1136/bmjopen-2015-010521

**Published:** 2016-03-23

**Authors:** Nigel Field, Philip Prah, Catherine H Mercer, Greta Rait, Michael King, Jackie A Cassell, Clare Tanton, Laura Heath, Kirstin R Mitchell, Soazig Clifton, Jessica Datta, Kaye Wellings, Anne M Johnson, Pam Sonnenberg

**Affiliations:** 1 Research Department of Infection and Population Health, UCL, London, UK; 2 Research Department of Primary Care and Population Health, UCL, London, UK; 3 Division of Psychiatry (Faculty of Brain Sciences), UCL, London, UK; 4 Division of Primary Care and Public Health, University of Brighton, Brighton, UK; 5 Department of Social and Environmental Health Research, London School of Hygiene and Tropical Medicine, London, UK; 6 UCL Medical School, UCL, London, UK

**Keywords:** Depression, Sexual health, Sexual health services

## Abstract

**Objective:**

To examine associations between sexual behaviour, sexual function and sexual health service use of individuals with depression in the British general population, to inform primary care and specialist services.

**Setting:**

British general population.

**Participants:**

15 162 men and women aged 16–74 years were interviewed for the third National Survey of Sexual Attitudes and Lifestyles (Natsal-3), undertaken in 2010–2012. Using age-adjusted ORs (aAOR), relative to a comparator group reporting no treatment or symptoms, we compared the sexual health of those reporting treatment for depression in the past year.

**Outcome measures:**

Sexual risk behaviour, sexual function, sexual satisfaction and sexual health service use.

**Results:**

1331 participants reported treatment for depression (5.2% men; 11.8% women). Relative to the comparator group, treatment for depression was associated with reporting 2 or more sexual partners without condoms (men aAOR 2.07 (95% CI 1.38 to 3.10); women 2.22 (1.68 to 2.92)), and concurrent partnerships (men 1.80 (1.18 to 2.76); women 2.06 (1.48 to 2.88)), in the past year. Those reporting depression treatment were more likely to be dissatisfied with their sex lives (men 2.32 (1.74 to 3.11); women 2.30 (1.89 to 2.79)), and to score in the lowest quintile on the Natsal-sexual function measure. They were also more likely to report a recent chlamydia test (men 1.92 (1.15 to 3.20)); women (1.27 (1.01 to 1.60)), and to have sought help regarding their sex life from a healthcare professional (men 2.92 (1.98 to 4.30); women (2.36 (1.83 to 3.04)), most commonly from a family doctor. Women only were more likely to report attending a sexual health clinic (1.91 (1.42 to 2.58)) and use of emergency contraception (1.98 (1.23 to 3.19)). Associations were broadly similar for individuals with depressive symptoms but not reporting treatment.

**Conclusions:**

Depression, measured by reported treatment, was strongly associated with sexual risk behaviours, reduced sexual function and increased use of sexual health services, with many people reporting help doing so from a family doctor. The sexual health of depressed people needs consideration in primary care, and mental health assessment might benefit people attending sexual health services.

**Strengths and limitations of this study**

- The major strength of this study lies in the comprehensive data collected on sexual behaviour, function and health service use linked to self-reported information on depression in a large probability sample. The National Survey of Sexual Attitudes and Lifestyles (Natsal-3) considered sexual health in its broadest sense according to the WHO-endorsed definition, and furthermore used the Natsal-SF measure, which takes account not only of sexual difficulties, but also the relationship context and overall levels of sexual satisfaction.
- The response rate for Natsal-3 (57.7%) was similar to other major social surveys, however, there may be systematic biases in who agrees to take part. To minimise this, we weighted the data to match the age, sex and regional profile of the British population according to the 2011 census. Once these weights had been applied, the Natsal-3 sample composition was generally comparable with the British population in terms of marital status, ethnic group, and self-reported general health.^19–21^
- Although the potential for reporting bias was minimised by using computer-assisted self-interview (CASI) and non-response weighting, our cross-sectional data should be interpreted with caution, and we note that causality cannot be assumed.
- In prioritising sexual health data collection, we relied on self-reporting of treatment for depression to an interviewer using computer-assisted personal interview (rather than objective clinical diagnosis or longer diagnostic tools), to identify a clinically meaningful group that would be relevant to family doctors and sexual health specialists. Although the prevalence and distribution of depression according to age, health and other factors matched data from other studies and data sources, we are not able to distinguish between pharmacological and psychological treatments for depression, the stage of treatment, or the extent to which treatment might be attenuating symptoms. We included a second measure of depression using the PHQ-2 screening tool, and found similar patterns of associations with a range of dependent sexual health variables for the group screening positive for recent depressive symptoms. This observation serves both to validate our findings and is suggestive of a population group with depressive symptoms and poor sexual health who might not be accessing, or adequately served by, healthcare services.
- The study is restricted to those reporting sexual experience who answered the Natsal-3 CASI, which corresponds to more than 95% of the British population.

## Introduction

In the UK, the estimated point prevalence of a major depressive episode among those aged 16–74 years is 2.6%, with less severe depression present in 9.1% of men and 13.6% of women.^1^ Over 90% of adults with depression are managed in primary care by their family doctor,^2^ and depression forms a large part of general practice (GP) case load, presenting in up to a quarter of GP attendees.^3^ ^4^ Psychological morbidity is also common in attendees of specialist sexual health clinics (ie, genitourinary medicine (GUM) clinics); one study found that over half the patients attending a London GUM clinic had experienced symptoms of depression, anxiety, or both in the past month,^5^ but this phenomenon is not well studied.

Depression has profound effects on quality of life, including sexual interest and behaviour.^6^ Studies, primarily in the USA and in adolescents, have shown associations between depression and sexual risk behaviours,^7–11^ and two North American studies extended these findings to adults.^12^ ^13^ Sexual dysfunction and depression frequently occur together, but the nature of their association is not well understood. Although reduced desire is most often reported, problems with arousal, resulting in vaginal dryness in women and erectile dysfunction in men, and absent or delayed orgasm are also common, and erectile dysfunction is sometimes used as a marker for depression.^14^ ^15^ Understanding the interaction between depression and sexual function is complicated by difficulty in determining the direction of causality,^16^ ^17^ the fact that patient groups are rarely treatment naïve, and by whether the condition or its treatment has the largest effect, since most antidepressants have undesired effects on sexual function.^18^

Few studies have investigated the overlap between sexual and mental well-being across the life course, and little is known about the sexual behaviour and sexual health service use of individuals with depression in the general population. This paper uses data from the third National Survey of Sexual Attitudes and Lifestyles (Natsal-3) to investigate the nature and strength of associations between depression and sexual behaviour, sexual function and sexual health service use in a representative sample of the British population aged 16–74 years.

## Methods

### Participants and procedures

Full details of the methods of Natsal-3 have been reported elsewhere.^19–21^ Briefly, households across Britain were selected using stratified probability sampling from which one eligible individual was selected at random and invited to participate. Altogether, 15 162 men and women aged 16–74 years were interviewed between September 2010 and August 2012. The overall response rate was 57.7%, and the cooperation rate was 65.8% (defined as the number of interviews completed from eligible addresses for which contact was made).^19^ ^22^ An anonymised data set has been deposited with the UK Data Service, persistent identified: 10.5255/UKDA-SN-7799–1, and the complete questionnaire and technical report are available on the Natsal website (http://www.natsal.ac.uk/home.aspx).

Participants were interviewed in their own homes by professional interviewers using computer-assisted personal interview. Participants were asked whether they had been diagnosed or treated for a range of chronic health conditions, listed on showcards,^23^ from which participants selected those they had experienced. In this way, a card was used to ask participants, ‘Are you currently taking any type of medicine prescribed by a doctor for depression?’. Participants subsequently completed a computer-assisted self-interview (CASI), which included a validated patient health questionnaire (PHQ-2), comprising two screening questions (‘During the past 2 weeks have you often been bothered by feeling down, depressed, or hopeless?’, and ‘During the past 2 weeks have you often been bothered by little interest or pleasure in doing things?’) to assess depressive symptoms in the past 2 weeks (each scored 0–3).^24^ Participants were deemed to have depressive symptoms if they had a total score of three or more, a cut-off that has been previously validated.^25^

**Figure 1:**
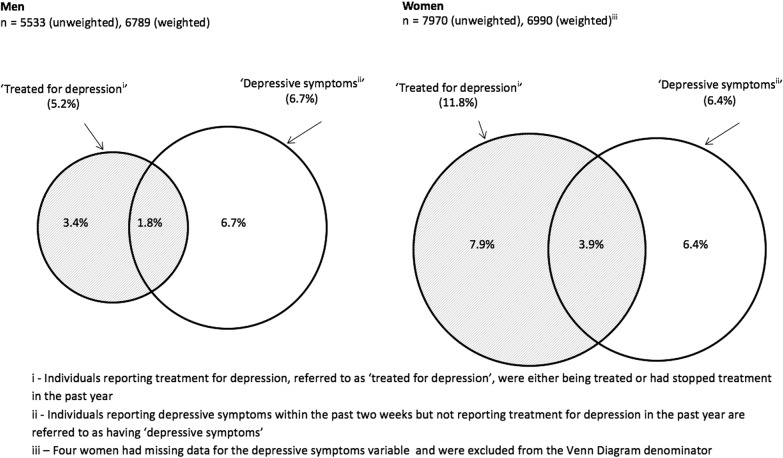
Proportional Venn diagrams showing the prevalence of ‘treatment for depression’ (i), and ‘depressive symptoms’ (ii) among men and women reporting one sexual partner, ever.

We identified a number of permutations in the groups that might be selected for analysis. To focus the analysis for a clinical practice audience, we selected three mutually exclusive groups of participants for comparison who might be readily defined in a clinical setting (figure 1), (1) individuals reporting treatment for depression, hereafter referred to as ‘treated for depression’, who were either being currently treated or had stopped treatment in the past year; (2) individuals reporting depressive symptoms within the past 2 weeks but not reporting treatment for depression in the past year, referred to as having ‘depressive symptoms’ and (3) a comparator group not treated for depression and without depressive symptoms. We included only sexually experienced participants, defined as those who reported having had one or more sexual partner by the time of their interview, because those reporting no previous sexual experience did not complete the CASI. We excluded from the analysis those reporting treatment from a healthcare professional in the past year for ‘any other mental health condition’ (12 men and 16 women).

The CASI included a range of other questions, including those asking about having sex without condoms, paying for sex, concurrent sexual partnerships, self-appraisal of sex life and sexual health service use, including reported clinic attendance, use of emergency contraception, and testing for sexually transmitted infections (STI).^20^ Our assessment of sexual function used the Natsal-SF,^26^ a validated measure comprising components on problems with sexual response, sexual function in a relationship context and self-appraisal of sex life. Wherever possible, the timeframes used in the analysis were those closest to the time frame for reporting treatment for depression (past year) or depressive symptoms (past 2 weeks).

### Statistical analyses

Analyses were carried out using the complex survey function of Stata V.13.1 (StataCorp LP, College Station, Texas, USA), accounting for the weighting, clustering and stratification of the Natsal-3 data. Survey weights were applied to adjust for unequal probability of selection and non-response to ensure the sample data were broadly representative of the British general population, according to the 2011 census, in terms of gender, age group and Government Office Region.^20^ ^21^

We estimated the prevalence with 95% CIs of reporting treatment for depression and depressive symptoms, by demographic and health factors. Our approach to multivariable analysis was guided by consideration of the obvious confounders, and we have been careful not to ‘over-adjust’. We feel that only age can be said to have an ‘obvious’ association with the behavioural outcomes that would confound the associations presented in this paper. We therefore used binary and ordinal logistic regression models to calculate age-adjusted ORs (aAORs) to examine associations between treatment for depression, or depressive symptoms in the absence of treatment, relative to the comparator group (those not treated for depression and without depressive symptoms), and (1) risky sexual behaviours, (2) sexual function and (3) sexual health service use.

## Results

### Prevalence of depression

Among 13 507 sexually experienced Natsal-3 participants aged 16–74 years (5533 men and 7974 women), the estimated prevalence of having received ‘treatment for depression’ in the past year was 5.2% (95% CI 4.6% to 5.9%) in men and 11.8% (11.0% to 12.7%) in women (tables 1 and 2; figure 1). A further 6.7% (6.0% to 7.5%) of men and 6.4% (5.8% to 7.1%) of women had ‘depressive symptoms’ in the past 2 weeks but did not report treatment in the past year, while 1.8% (1.5% to 2.3%) of men and 3.9% (3.5% to 4.5%) of women reported treatment for depression and had depressive symptoms. In both men and women, treatment for depression was highest among those aged 35–64 years, whereas depressive symptoms were most common in those aged 16–24 years.

**Table 1:** Variations in the prevalence of reporting treatment for depression and depressive symptoms by key demographic and health characteristics, men

	Men								
	Not treated and without depressive symptoms		Treatment within the past year			Depressive symptoms (without treatment)			Unweighted, weighted denominator
	Per cent	CI (%)	Per cent	CI (%)	aAOR*	Per cent	CI (%)	aAOR†	
Overall									
	88.1	(87.1 to 89.0)	5.2	(4.6 to 5.9)	–	6.7	(6.0 to 7.5)	–	5533, 6789
Age group									
16–24	88.1	(86.1 to 89.9)	2.6	(1.8 to 3.7)	1.00	9.3	(7.7 to 11.1)	1.00	1346, 984
25–34	88.5	(86.5 to 90.2)	4.4	(3.4 to 5.6)	1.66 (1.06 to 2.58)	7.1	(5.7 to 8.9)	0.77 (0.56 to 1.05)	1398, 1256
35–44	87.4	(84.8 to 89.7)	6.8	(5.3 to 8.8)	2.61 (1.65 to 4.15)	5.8	(4.2 to 7.8)	0.63 (0.43 to 0.92)	762, 1357
45–54	88.2	(85.7 to 90.3)	6.2	(4.7 to 8.2)	2.37 (1.47 to 3.80)	5.5	(4.1 to 7.4)	0.60 (0.41 to 0.87)	728, 1316
55–64	85.3	(82.2 to 87.9)	6.9	(5.2 to 9.1)	2.73 (1.70 to 4.37)	7.8	(5.8 to 10.4)	0.87 (0.60 to 1.26)	698, 1097
65–74	92.4	(89.9 to 94.3)	2.8	(1.8 to 4.3)	1.01 (0.55 to 1.84)	4.8	(3.2 to 7.0)	0.49 (0.31 to 0.78)	601, 778
Relationship status									
Living with a partner	91.0	(89.8 to 92.0)	3.6	(3.0 to 4.4)	1.00	5.4	(4.6 to 6.4)	1.00	2912, 4628
Steady relationship (not cohabiting)	86.7	(84.0 to 89.0)	6.3	(4.6 to 8.7)	2.05 (1.35 to 3.09)	7.0	(5.3 to 9.0)	1.26 (0.89 to 1.79)	933, 748
Previously in a live-in partnership	73.8	(70.3 to 77.1)	15.5	(12.7 to 18.6)	5.21 (3.82 to 7.11)	10.7	(8.6 to 13.2)	2.46 (1.81 to 3.34)	754, 674
Not in a steady relationship (never cohabited)	85.0	(82.2 to 87.5)	4.5	(3.2 to 6.3)	1.54 (0.98 to 2.41)	10.5	(8.4 to 12.9)	1.90 (1.34 to 2.69)	894, 702
Ended a cohabiting relationship, past year									
No	88.8	(87.8 to 89.7)	4.7	(4.1 to 5.4)	1.00	6.5	(5.8 to 7.3)	1.00	5094, 6398
Yes	72.0	(65.6 to 77.7)	16.1	(11.4 to 22.1)	4.47 (2.93 to 6.83)	11.9	(8.2 to 17.0)	2.14 (1.38 to 3.31)	277, 237
Sexual identity									
Heterosexual/straight	88.4	(87.4 to 89.3)	5.0	(4.4 to 5.6)	1.00	6.6	(5.9 to 7.4)	1.00	5349, 6599
Gay	81.6	(71.2 to 88.8)	14.7	(8.5 to 24.2)	3.32 (1.75 to 6.30)	3.8	(1.3 to 10.5)	0.60 (0.20 to 1.79)	106, 102
Bisexual	70.7	(56.1 to 82.1)	10.8	(5.2 to 21.0)	2.74 (1.20 to 6.27)	18.5	(9.5 to 33.0)	3.52 (1.56 to 7.94)	61, 71
Other	NA	NA	NA	NA	NA	NA	NA	NA	13, 11
Academic qualifications‡									
No academic qualifications	83.9	(81.4 to 86.1)	7.4	(6.0 to 9.2)	1.00	8.7	(7.0 to 10.8)	1.00	1153, 1509
Academic qualifications typically gained at age 16 years	87.9	(86.1 to 89.4)	5.7	(4.6 to 6.9)	0.72 (0.51 to 1.03)	6.5	(5.3 to 7.9)	0.61 (0.43 to 0.85)	1921, 2322
Studying for/attained further academic qualifications	90.2	(88.8 to 91.5)	4.0	(3.2 to 4.9)	0.50 (0.34 to 0.72)	5.8	(4.8 to 7.0)	0.51 (0.37 to 0.72)	2851, 3361
Deprivation quintile									
1 (least deprived)	91.4	(89.4 to 93.0)	3.2	(2.3 to 4.5)	1.00	5.4	(4.2 to 6.9)	1.00	1096, 1428
2	90.9	(88.8 to 92.7)	3.9	(2.7 to 5.4)	1.21 (0.73 to 2.02)	5.2	(3.9 to 7.0)	0.97 (0.64 to 1.47)	1109, 1459
3	89.1	(86.8 to 91.1)	5.0	(3.8 to 6.6)	1.65 (1.04 to 2.61)	5.8	(4.4 to 7.7)	1.10 (0.73 to 1.65)	1081, 1320
4	85.9	(83.3 to 88.1)	6.2	(4.8 to 7.9)	2.16 (1.38 to 3.38)	7.9	(6.3 to 10.0)	1.53 (1.04 to 2.25)	1098, 1337
5 (most deprived)	82.3	(79.7 to 84.7)	8.1	(6.6 to 10.0)	2.99 (1.95 to 4.58)	9.5	(7.7 to 11.7)	1.91 (1.32 to 2.76)	1149, 1245
Self-reported health									
Fair/good/very good	89.5	(88.5 to 90.3)	4.3	(3.7 to 5.0)	1.00	6.2	(5.6 to 7.0)	1.00	5338, 6545
Bad/very bad	52.0	(43.9 to 59.9)	29.4	(23.1 to 36.7)	12.07 (8.07 to 18.06)	18.6	(13.0 to 25.9)	6.19 (3.85 to 9.95)	193, 241
Drink >6/8 units on one occasion—weekly									
No	88.2	(87.0 to 89.3)	5.2	(4.5 to 6.0)	1.00	6.6	(5.7 to 7.5)	1.00	3969, 4997
Yes	88.6	(86.6 to 90.4)	5.0	(3.8 to 6.4)	0.97 (0.71 to 1.33)	6.4	(5.1 to 8.0)	0.94 (0.71 to 1.24)	1209, 1371
Taken recreational drugs, past year									
No	89.4	(88.4 to 90.3)	4.7	(4.1 to 5.4)	1.00	5.9	(5.2 to 6.7)	1.00	4426, 5746
Yes	80.8	(77.7 to 83.5)	8.2	(6.5 to 10.4)	2.47 (1.76 to 3.47)	11.0	(8.9 to 13.5)	2.02 (1.50 to 2.71)	1098, 1031
Current cigarette smoker									
No	89.9	(88.8 to 91.0)	4.0	(3.4 to 4.8)	1.00	6.0	(5.2 to 7.0)	1.00	3886, 4998
Yes	83.0	(81.0 to 84.9)	8.4	(7.1 to 10.0)	2.40 (1.83 to 3.14)	8.5	(7.2 to 10.1)	1.48 (1.16 to 1.88)	1647, 1791

All participants who have had at least one opposite-sex or same-sex partner, ever.

*OR for the outcome of reporting treatment for depression compared to not treated for depression—screened negative.

**†**OR for the outcome of reporting screened positive for depression compared to not treated for depression—screened negative.

**‡**Participants aged 17+ years.

aAOR, age-adjusted OR; NA, non-applicable due to too few participants.

**Table 2:** Variations in the prevalence of reporting treatment for depression and depressive symptoms by key demographic and health characteristics, women

	Women								
	Not treated and without depressive symptoms		Treatment within the past year			Depressive symptoms (without treatment)			Unweighted, weighted denominator
	Per cent	CI (%)	Per cent	CI (%)	aAOR*	Per cent	CI (%)	aAOR†	
Overall									
	81.7	(80.7 to 82.7)	11.8	(11.0 to 12.7)	–	6.4	(5.8 to 7.1)	–	7974, 6995
Age group									
16–24	80.8	(78.6 to 82.8)	9.2	(7.7 to 10.8)	1.00	10.1	(8.6 to 11.7)	1.00	1688, 936
25–34	82.5	(80.8 to 84.1)	11.7	(10.4 to 13.1)	1.25 (1.00 to 1.57)	5.8	(4.8 to 6.9)	0.56 (0.43 to 0.73)	2315, 1278
35–44	80.5	(77.9 to 82.8)	13.6	(11.7 to 15.8)	1.49 (1.16 to 1.93)	5.9	(4.6 to 7.6)	0.59 (0.43 to 0.82)	1153, 1385
45–54	78.7	(75.8 to 81.4)	14.8	(12.6 to 17.4)	1.66 (1.28 to 2.17)	6.4	(5.0 to 8.4)	0.66 (0.48 to 0.91)	1044, 1364
55–64	83.0	(80.4 to 85.4)	12.0	(10.0 to 14.3)	1.28 (0.98 to 1.67)	5.0	(3.7 to 6.7)	0.48 (0.33 to 0.70)	967, 1167
65–74	86.7	(83.9 to 89.1)	7.2	(5.5 to 9.4)	0.73 (0.51 to 1.04)	6.1	(4.5 to 8.2)	0.57 (0.40 to 0.80)	807, 865
Relationship status									
Living with a partner	84.3	(83.1 to 85.5)	10.2	(9.2 to 11.2)	1.00	5.5	(4.7 to 6.3)	1.00	4306, 4647
Steady relationship (not cohabiting)	79.6	(77.0 to 82.1)	13.0	(11.0 to 15.3)	1.26 (1.00 to 1.59)	7.3	(5.9 to 9.2)	1.26 (0.92 to 1.71)	1341, 779
Previously in a live-in partnership	73.6	(71.0 to 76.0)	18.4	(16.4 to 20.7)	2.13 (1.77 to 2.57)	8.0	(6.6 to 9.6)	1.75 (1.34 to 2.28)	1558, 1108
Not in a steady relationship (never cohabited)	78.6	(75.0 to 81.8)	10.4	(8.1 to 13.2)	1.00 (0.73 to 1.37)	11.0	(8.8 to 13.7)	1.84 (1.35 to 2.50)	719, 426
Ended a cohabiting relationship, past year									
No	82.3	(81.3to 83.3)	11.5	(10.6to 12.4)	1.00	6.2	(5.6 to 6.9)	1.00	7219, 6562
Yes	68.3	(63.4 to 72.8)	21.8	(18.0 to 26.2)	2.28 (1.76 to 2.96)	9.9	(7.0to 13.8)	1.77 (1.18 to 2.67)	484, 256
Sexual identity									
Heterosexual/straight	82.1	(81.0 to 83.0)	11.6	(10.8 to 12.5)	1.00	6.3	(5.7 to 7.0)	1.00	7731, 6811
Gay	72.7	(61.0 to 82.0)	16.1	(9.1 to 27.1)	1.56 (0.80 to 3.05)	11.1	(5.6 to 20.9)	1.91 (0.88 to 4.12)	85, 70
Bisexual	68.7	(58.9 to 77.1)	24.3	(16.6 to 34.1)	2.46 (1.50 to 4.04)	7.0	(4.0 to 11.8)	1.15 (0.63 to 2.12)	134, 90
Other	NA	NA	NA	NA	NA	NA	NA	NA	16, 15
Academic qualifications‡									
No academic qualifications	77.1	(74.6 to 79.4)	14.9	(13.0 to 17.0)	1.00	8.0	(6.6 to 9.8)	1.00	1562, 1531
Academic qualifications typically gained at age 16 years	79.8	(78.0 to 81.5)	14.0	(12.6 to 15.6)	0.81 (0.66 to 1.01)	6.2	(5.2 to 7.3)	0.61 (0.45 to 0.83)	2851, 2509
Studying for/attained further academic qualifications	85.4	(84.0 to 86.7)	9.2	(8.1 to 10.5)	0.47 (0.37 to 0.60)	5.4	(4.6 to 6.3)	0.45 (0.33 to 0.62)	3957, 3261
Deprivation quintile									
1 (least deprived)	86.4	(84.4 to 88.1)	8.9	(7.4 to 10.6)	1.00	4.7	(3.7 to 6.0)	1.00	1485, 1442
2	86.5	(84.5 to 88.3)	9.2	(7.7 to 10.9)	1.03 (0.78 to 1.36)	4.3	(3.3 to 5.6)	0.91 (0.63 to 1.32)	1564, 1456
3	83.6	(81.3 to 85.6)	10.1	(8.6 to 11.9)	1.18 (0.90 to 1.55)	6.3	(4.9 to 8.0)	1.34 (0.94 to 1.93)	1561, 1373
4	77.7	(75.2 to 80.0)	14.3	(12.3 to 16.5)	1.79 (1.37 to 2.33)	8.0	(6.6 to 9.7)	1.83 (1.31 to 2.55)	1650, 1389
5 (most deprived)	73.9	(71.4 to 76.2	17.2	(15.2 to 19.3)	2.27 (1.77 to 2.90)	8.9	(7.5 to 10.6)	2.13 (1.53 to 2.95)	1714, 1336
Self-reported health									
Fair/good/very good	83.4	(82.4 to 84.4)	10.6	(9.8 to 11.4)	1.00	6.0	(5.4 to 6.6)	1.00	7666, 6689
Bad/very bad	45.0	(38.6 to 51.7	39.3	(33.2 to 45.8)	7.41 (5.48 to 10.01)	15.6	(11.5 to 21.0)	5.89 (3.89 to 8.92)	308, 305
Drink >6/8 units on one occasion—weekly									
No	82.5	(81.4 to 83.5)	11.3	(10.4 to 12.2)	1.00	6.3	(5.6 to 7.0)	1.00	6179, 5485
Yes	79.7	(76.3 to 82.7)	14.6	(12.0 to 17.6)	1.33 (1.05 to 1.70)	5.7	(4.1 to 7.9)	0.91 (0.62 to 1.32)	930, 766
Taken recreational drugs, past year									
No	82.3	(81.3 to 83.3)	11.3	(10.5 to 12.2)	1.00	6.3	(5.7 to 7.0)	1.00	7219, 6508
Yes	73.8	(69.7 to 77.4)	18.4	(15.0 to 22.3)	1.83 (1.39 to 2.41)	7.8	(6.1to 10.1)	1.20 (0.88 to 1.62)	738, 472
Current cigarette smoker									
No	84.0	(82.9 to 85.0)	10.0	(9.1 to 10.9)	1.00	6.0	(5.3 to 6.8)	1.00	5759, 5331
Yes	74.6	(72.4 to 76.6)	17.8	(16.0 to 19.7)	2.01 (1.71 to 2.36)	7.7	(6.5 to 9.0)	1.38 (1.11 to 1.72)	2215, 1664

All participants who have had at least one opposite-sex or same-sex partner, ever.

*OR for the outcome of reporting treatment for depression compared to not treated for depression—screened negative.

†OR for the outcome of reporting screened positive for depression compared to not treated for depression—screened negative.

‡Participants aged 17+ years.

aAOR, age-adjusted OR; NA, non-applicable due to too few participants.

### Treatment for depression

After adjusting for age, treatment for depression was more common among men and women reporting a previous live-in partnership but currently single, and those currently in a non-cohabiting steady relationship (tables 1 and 2). There were 16.1% of men and 21.8% of women who reported ending a cohabiting relationship in the past year who had received treatment for depression in the same period, compared with 5.2% of men and 11.8% of women in the comparator group without depression (aAORs 4.47 (2.93 to 6.83) for men and 2.28 (1.76 to 2.96) for women). Treatment for depression was also more likely to be reported by those with lower academic attainment, those living in more deprived areas, as measured by area-level deprivation, and by men self-identifying as gay or bisexual, and by bisexual women.

Nearly one-third of men and nearly one-fifth of women self-reporting bad or very bad general health status reported treatment for depression, and the association remained strong after adjusting for age (aAORs 12.07 (8.08 to 18.06) for men and 7.41 (5.48 to 10.01) for women) (tables 1 and 2). Treatment for depression was also more likely among men and women who smoked and those who had taken recreational drugs in the past year. The association with binge drinking was less clear; for women only, treatment for depression was associated with reporting weekly consumption of six or more units of alcohol on any one occasion.

#### Sexual behaviour and STI risk

Men and women treated for depression reported fewer occasions of sexual activity in the past 4 weeks than the comparator group (table 3). After adjusting for age, reporting treatment for depression remained strongly associated with sexual behaviours that are markers for STI transmission. Treatment for depression was associated with reporting sex without condoms with two or more partners in the past year (aAOR 2.07 (1.38 to 3.10) in men and aAOR 2.22 (1.68 to 2.92) in women), and a same-sex partner in the past 5 years (aAOR 2.70 (1.57 to 4.62) in men and aAOR 2.02 (1.44 to 2.83) in women). In women, treatment for depression was also associated with partnership concurrency in the past 5 years (aAOR 2.01 (1.59 to 2.55)), the perception that a partner had had sex with someone else in the past 5 years (aAOR 1.55 (1.28 to 1.86)), and a higher self-perceived risk of STIs (aAOR 1.99 (1.26 to 3.15)).

**Table 3:** Sexual behaviour and STI risk in those reporting treatment for depression, by gender

	Men		Women	
	Not treated and without depressive symptoms	Treatment within the past year	Not treated and without depressive symptoms	Treatment within the past year
Unweighted, weighted denominator	4809, 5981	319, 353	6413, 5718	1012, 828
2+ heterosexual/same-sex occasions of sexual intercourse, past 4 weeks*				
Per cent	68.7	55.9	66.1	57.7
OR (95% CI)	1.00	**0.58 (0.42 to 0.79)**	1.00	**0.70 (0.58 to 0.85)**
aAOR (95% CI)	1.00	**0.58 (0.43 to 0.80)**	1.00	**0.68 (0.56 to 0.82)**
2+ heterosexual partners, past year				
Per cent	15.1	18.0	8.7	13.4
OR (95% CI)	1.00	1.24 (0.90 to 1.71)	1.00	**1.63 (1.32 to 2.01)**
aAOR (95% CI)	1.00	**1.48 (1.05 to 2.09)**	1.00	**1.90 (1.52 to 2.38)**
Same-sex partner, past 5 years				
Per cent	2.5	6.3	2.8	5.4
OR (95% CI)	1.00	**2.59 (1.52 to 4.41)**	1.00	**1.94 (1.39 to 2.70)**
aAOR (95% CI)	1.00	**2.70 (1.57 to 4.62)**	1.00	**2.02 (1.44 to 2.83)**
2+ heterosexual/same-sex partners without using a condom, past year				
Per cent	7.4	12.2	4.4	8.0
OR (95% CI)	1.00	**1.74 (1.18 to 2.57)**	1.00	**1.90 (1.46 to 2.48)**
aAOR (95% CI)	1.00	**2.07 (1.38 to 3.10)**	1.00	**2.22 (1.68 to 2.92)**
Paid for heterosexual/same sex, past year				
Per cent	1.1	2.2	0.1	0.0
OR (95% CI)	1.00	2.11 (0.76 to 5.89)	NA	NA
aAOR (95% CI)	1.00	2.11 (0.76 to 5.92)	NA	NA
Concurrent partnerships, past 5 years				
Per cent	15.0	16.9	7.1	12.0
OR (95% CI)	1.00	1.15 (0.83 to 1.60)	1.00	**1.79 (1.43 to 2.25)**
aAOR (95% CI)	1.00	1.27 (0.90 to 1.78)	1.00	**2.01 (1.59 to 2.55)**
Know/ perceive their most recent partner had sex with someone else, past 5 years†				
Per cent	22.8	28.5	22.2	30.6
OR (95% CI)	1.00	1.35 (0.95 to 1.91)	1.00	**1.55 (1.28 to 1.87)**
aAOR (95% CI)	1.00	1.36 (0.95 to 1.93)	1.00	**1.55 (1.28 to 1.87)**
Diagnosed with a STI, past year				
Per cent	0.9	1.1	0.8	1.1
OR (95% CI)	1.00	1.34 (0.44 to 4.07)	1.00	1.33 (0.72 to 2.44)
aAOR (95% CI)	1.00	1.80 (0.59 to 5.54)	1.00	1.58 (0.86 to 2.90)
Self-perceived risk of STI: Quite a lot or greater				
Per cent	3.4	4.1	2.1	3.9
OR (95% CI)	1.00	1.20 (0.69 to 2.09)	1.00	**1.93 (1.22 to 3.03)**
aAOR (95% CI)	1.00	1.34 (0.77 to 2.35)	1.00	**1.99 (1.26 to 3.15)**

All participants who have had at least one opposite-sex or same-sex partner, ever (denominators may vary across variables due to item non-response).

Bold typeface highlights results where the confidence intervals exclude 1.0.

*Denominator restricted to those reporting at least one heterosexual or same-sex partner in the last year (men: not treated and without depressive symptoms—48 095 981; treated for depression—319 353; women: not treated and without depressive symptoms—64 135 718; treated for depression—1 012 828).

†Denominator restricted to those reporting a sexual partnership in the past 5 years (Men: Not treated and without depressive symptoms – 3955, 3912; Treated for depression – 241, 197 | Women: Not treated and without depressive symptoms – 5273, 3657; Treated for depression – 824, 465).

aAOR, age-adjusted OR; STI, sexually transmitted infections; NA, non-applicable due to too few participants.

#### Sexual function

Among those with at least one sexual partner in the past year (5054 men and 7332 women), individuals reporting treatment for depression were much more likely, after adjusting for age, than those without any depression to have ‘low sexual function’ as measured using the Natsal-SF score: for men treated for depression, the aAOR was 2.55 (1.85 to 3.52) and in women was 2.64 (2.16 to 3.23).

Among those treated for depression, 29.5% of men and 22.4% of all women reported being dissatisfied with their sex life, compared to 15.1% of men and 11.2% of women without depression, and the association remained after adjusting for age (table 4). There were strong associations between depression and other specific indicators of low sexual function, and the adjusted ORs were of similar magnitude when comparing men and women across the different variables. We found similar associations between depression and lacking interest in sex, and having trouble reaching orgasm. In all cases, the associations were not affected by adjusting for age (data shown) or relationship status (data not shown).

**Table 4:** Sexual function of those reporting treatment for depression, by gender

	Men		Women	
	Not treated and without depressive symptoms	Treatment within the past year	Not treated and without depressive symptoms	Treatment within the past year
Unweighted, weighted denominator	4750, 5914	314, 348	6334, 5650	998, 814
Lowest quintile of sexual function (ref)				
Per cent	17.3	34.8	16.2	33.1
OR (95% CI)	1.00	**2.55 (1.85 to 3.51)**	1.00	**2.55 (2.09 to 3.12)**
aAOR (95% CI)	1.00	**2.55 (1.85 to 3.52)**	1.00	**2.64 (2.16 to 3.23)**
Components of the Natsal-SF score* (ref)				
Have experienced problems, for 3+ months, past year				
Per cent	39.3	62.1	46.7	68.5
OR (95% CI)	1.00	**2.54 (1.86 to 3.45)**	1.00	**2.48 (2.06 to 3.00)**
aAOR (95% CI)	1.00	**2.53 (1.85 to 3.45)**	1.00	**2.52 (2.09 to 3.04)**
Lacked interest in having sex, for 3+ months, past year				
Per cent	13.0	29.0	30.0	50.9
OR (95% CI)	1.00	**2.74 (1.95 to 3.87)**	1.00	**2.42 (2.02 to 2.91)**
aAOR (95% CI)	1.00	**2.74 (1.94 to 3.86)**	1.00	**2.46 (2.05 to 2.96)**
No orgasm or took a long time to reach orgasm despite arousal, for3+ months, past year				
Per cent	8.2	20.0	14.2	24.9
OR (95% CI)	1.00	**2.80 (1.91 to 4.12)**	1.00	**2.00 (1.62 to 2.48)**
aAOR (95% CI)	1.00	**2.80 (1.90 to 4.12)**	1.00	**2.00 (1.61 to 2.48)**
Trouble achieving/maintaining erections, for 3+ months, past year				
Per cent	12.2	20.0		
OR (95% CI)	1.00	**1.80 (1.22 to 2.66)**		
aAOR (95% CI)	1.00	**1.86 (1.25 to 2.78)**		
Disagree/ disagree strongly to the statement: “I feel satisfied with my sex life"				
Per cent	15.1	29.5	11.2	22.4
OR (95% CI)	1.00	**2.35 (1.76 to 3.14)**	1.00	**2.28 (1.88 to 2.77)**
aAOR (95% CI)	1.00	**2.32 (1.74 to 3.11)**	1.00	**2.30 (1.89 to 2.79)**
Perceive a health condition/disability has affected sexual activity/enjoyment, past year				
Per cent	13.5	45.7	12.8	36.7
OR (95% CI)	1.00	**5.40 (4.10 to 7.09)**	1.00	**3.94 (3.31 to 4.70)**
aAOR (95% CI)	1.00	**5.74 (4.29 to 7.68)**	1.00	**3.96 (3.32 to 4.72)**
Perceive medications taken have limited sexual activity/enjoyment, past year				
Per cent	6.2	36.5	3.8	24.5
OR (95% CI)	1.00	**8.69 (6.39 to 11.82)**	1.00	**8.15 (6.44 to 10.31)**
aAOR (95% CI)	1.00	**10.28 (7.22 to 14.63)**	1.00	**8.15 (6.44 to 10.31)**
Taken any medicine/pills to assist sexual performance, past year				
Per cent	6.2	10.6	0.7	1.7
OR (95% CI)	1.00	**1.80 (1.16 to 2.80)**	1.00	**2.54 (1.33 to 4.88)**
aAOR (95% CI)	1.00	**1.77 (1.12 to 2.81)**	1.00	**2.62 (1.39 to 4.93)**

All participants who have had at least one opposite-sex or same-sex partner, ever (denominators may vary across variables due to item non-response).

Bold typeface highlights results where the confidence intervals exclude 1.0.

*All participants who have had at least one opposite-sex or same-sex partner, past year.

aAOR, age-adjusted ORo; Natsal, National Survey of Sexual Attitudes and Lifestyles.

There were strong associations between treatment for depression and reporting that a health condition had affected sexual activity, although we do not know whether the condition was depression. Similarly, 36.5% of men and 24.5% of women who reported taking medications that had limited their sexual activity also reported treatment for depression (aAORs 10.28 (7.22 to 14.63) for men and 8.15 (6.44 to 10.31) for women) (table 4). Those treated for depression were more likely to report taking drugs to assist their sexual performance (aAORs 1.77 (1.12 to 2.81) for men and 2.62 (1.39 to 4.93) for women).

#### Use of sexual health services

For men, although there was no association between reporting treatment for depression and sexual health clinic attendance in the past year, those treated for depression were more likely to report a recent chlamydia test (aAOR 1.92 (1.15 to 3.20), and to have sought help regarding their sex life in the past year from a healthcare professional (aAOR 2.92 (1.98 to 4.30) (table 5). The source of professional help was their GP/family doctor for 70% of these men, a psychiatrist/psychologist for 20%, and another healthcare provider for 10%.

**Table 5:** Sexual health service use of those reporting treatment in the past year, by gender

	Men		Women	
	Not treated and without depressive symptoms	Treatment within the past year	Not treated and without depressive symptoms	Treatment within the past year
Unweighted, weighted denominator	4809, 5981	319, 353	6413, 5718	1012, 828
Attended a sexual health (GUM) clinic, past year				
Per cent	4.2	2.8	4.4	6.5
aAOR (95% CI)	1.00	0.94 (0.51 to 1.73)	1.00	**1.91 (1.42** to **2.58)**
AOR* (95% CI)	1.00	0.64 (0.33 to 1.25)	1.00	**1.60 (1.15** to **2.24)**
Had a chlamydia test, past year†				
Per cent	15.6	18.0	25.1	25.7
aAOR (95% CI)	1.00	**1.92 (1.15** to **3.20)**	1.00	**1.27 (1.01** to **1.60)**
AOR* (95% CI)	1.00	1.67 (0.97 to 2.89)	1.00	1.18 (0.93 to 1.49)
Had a blood test for HIV, past year				
Per cent	3.4	3.4	5.3	4.5
aAOR (95% CI)	1.00	1.16 (0.65 to 2.10)	1.00	0.93 (0.68 to 1.27)
AOR* (95% CI)	1.00	0.96 (0.50 to 1.84)	1.00	0.86 (0.63 to 1.18)
You or your partner used emergency contraception, past year				
Per cent	2.3	2.0	1.5	2.5
aAOR (95% CI)	1.00	1.17 (0.52 to 2.61)	1.00	**1.98 (1.23** to **3.19)**
AOR* (95% CI)	1.00	1.01 (0.45 to 2.26)	1.00	**1.71 (1.07** to **2.74)**
Sought professional help regarding your sex life, past year				
Per cent	6.4	16.6	5.7	12.5
aAOR (95% CI)	1.00	**2.92 (1.98** to **4.30)**	1.00	**2.36 (1.83** to **3.04)**
AOR* (95% CI)	1.00	**2.89 (1.95** to **4.28)**	1.00	**2.36 (1.83** to **3.04)**

All participants who have had at least one opposite-sex or same-sex partner, ever (denominators may vary across variables due to item non-response).

Bold typeface highlights results where the confidence intervals exclude 1.0.

*Adjusted for age and 2+ partners without using a condom, past year.

†Participants aged 16–44 years.

aAOR, age-adjusted OR; GUM, genitourinary medicine.

Women treated for depression were more likely to report attending a sexual health clinic (aAORs 1.91 (1.42 to 2.58)), having a recent chlamydia test (aAOR 1.27 (1.01 to 1.60)), and use of emergency contraception in the past year (aAOR 1.98 (1.23 to 3.19), and were also more likely to have sought professional help regarding their sex life (aAOR 2.36 (1.83 to 3.04)). GPs/family doctors were the source of sexual advice in 70% of these women, and a sexual health clinic, psychiatrist/psychologist or relationship counsellor were each reported by 10%.

### Depressive symptoms

In a second analysis, using the same statistical approach, we investigated the sexual health of participants reporting depressive symptoms in the past 2 weeks, but not treatment in the previous year. Compared to those reporting treatment for depression, the overall patterns of associations were similar for individuals with depressive symptoms, although the strength of associations was generally weaker, except for sexual function, where the associations were of a similar magnitude (see online supplementary appendix tables 1–3). Some differences were noted for men reporting depressive symptoms; for example, we observed significant associations with reporting of current partnerships, with the perception that a partner had had sex with someone else in the past 5 years, and with a higher self-perceived risk of STIs; associations that were not significant for men reporting treatment for depression. On the contrary, there were not associations for health service use for men, and only an association with seeking professional help for women with depressive symptoms.

10.1136/bmjopen-2015-010521.supp1 Supplementary appendix

## Discussion

### Statement of principal findings

This study investigated the sexual health of people with a history of treatment for depression or depressive symptoms in a representative sample of the sexually experienced British general population aged 16–74 years. Individuals treated for depression, or with depressive symptoms, were more likely to report a range of sexual health difficulties. Those treated for depression were more likely to report behaviours linked to STI acquisition and/or transmission^27^ and depression was also strongly associated with low sexual function among men and women. For example, around two-thirds of those treated for depression reported problems with their sex lives, but only around 15% had sought professional help (and in most cases this was their GP/family doctor). Age adjustment made little difference to these findings, suggesting that these problems are experienced at all ages. Women treated for depression, but not men, were more likely to report accessing sexual health clinics, suggesting that depressed men in particular might be inadequately served by sexual health services.

We observed similar associations for a second group in the population, those with current depressive symptoms but no history of being treated for depression in the past year, for whom there was also evidence of being at greater risk of STIs, and having worse sexual function. This group is likely to include individuals with unmet mental health needs, who might be readily identifiable within a clinic population using the PHQ-2 screening tool. It was noticeable that this group was not more likely than those without reported symptoms or treated depression to use health services, suggesting coexiting unmet needs of mental health and sexual health.

These data provide valuable information to GPs, as well as to sexual health specialists, which may be used to improve the sexual health of patients with depression.

### Comparison with other studies

Our study is unique in taking a broad-based approach to understanding the relationship between depression and sexual health at a general population level across the life course, and considering three key components of sexual health in people with depression: behaviour, function and health service use. This study considerably extends our previous work, where we showed that poor health, including depression, is independently associated with reduced sexual well-being at all ages in Britain.^23^ ^28^ Other studies have reported on behaviour and function in isolation, and not considered service use. There is good evidence to suggest that depression is associated with increased sexual risk-taking in adolescent populations in North America. For example, the US National Longitudinal Adolescent Health Study found that depression was associated with reporting multiple partners, but was not associated with condom use (key to STI transmission),^9^ but there is a lack of consensus about the direction of association.^29^ ^30^ Two studies are noteworthy in adult populations, again from North America. Pratt *et al*, found associations in US women aged 20–59 years between depressive symptoms (scored using the nine-question PHQ-9) and age at first sex, numbers of sexual partners in the past year, and infection with Herpes Simplex Virus-2, but there were no associations in men.^12^ Chen *et al*,^13^ used a 27-question approach to determine major depressive episodes in the past 12 months in a sample of Canadians aged 15–49 years, and found an association between a history of STIs and depressive symptoms in women and men younger than 35 years. By contrast, our study focuses on a group reporting clinical treatment, and found associations in both men and women across the life course. The differences in our findings might, in part, be due to our survey methodology, which has been specifically designed, with refinement over two decades, to collect the most accurate data about sensitive sexual behaviours.

Other surveys in the UK have shown that mental health symptoms are predictive of impaired sexual interest and function in men and women,^31^ and a recent systematic review also made this link.^32^ Studies in which partners have been included confirm the poorer sexual function, at least in men,^33^ who report fewer and weaker nocturnal erections.^34^ The likely mechanisms behind these associations are complex. They include a cognitive bias where depressed people experience lower satisfaction with sex as well as other activities;^35^ higher risk of metabolic syndrome in depressed people with the cardiovascular sequelae including sexual dysfunction;^36^ neuroendocrine changes, and the effects of treatment.^37^ Up to two-thirds of people treated with selective serotonin reuptake inhibitors report sexual side effects,^38^ and such side effects are often a reason for ceasing to take the medication. Furthermore, antidepressants are often prescribed off-license to treat premature ejaculation, and their inhibitory sexual effects are well known.^39^ Our findings were striking in the strength of associations seen for reduced sexual function, and the high proportion reporting that health conditions and medications had affected their sex lives. This extended to specific symptoms, and we found that more than three in five men, and more than two in three women reporting treatment for depression had also experienced sexual problems for more than 3 months in the past year. Our study is also unique in including sexual health associations for a group reporting depressive symptoms but no treatment. We observed similar proportions of those reporting depressive symptoms but no treatment had experienced sexual problems, which suggests that depression rather than its treatment is more important in driving this association.

We know that individuals with sexual health problems often do not seek help,^40^ ^41^ and we also know that attending specialist sexual health clinics can evoke a range of psychological symptoms and concerns.^42^ However, there are few data available addressing the important question about use of sexual health service in people with depression, and our study adds substantially to the literature in this respect.

### Implications for policy and practice

Most depression is managed in primary care,^2^ and most people with sexual function problems initially seek professional help from their family doctor.^43^ In Britain, many sexual health services are being moved into community and other primary care settings, which provides an opportunity to align these areas of healthcare.^44^ ^45^ We have considered two groups of individuals who may present to primary care, those receiving treatment for depression and those with depressive symptoms but not recently treated, and we observed evidence of poor sexual health in both. Depressed patients may therefore derive benefit from holistic assessment of sexual health and mental health,^9^ and by extension, appropriate and successful treatment of depression might reduce sexual risk-taking, and improve sexual function.

Our finding that men identifying as gay, and those reporting same-sex partners, and women identifying as bisexual and those reporting same-sex partners, were more likely to report treatment for depression, is consistent with the literature on lesbian, gay and bisexual (LGB) people,^46–49^ who are at higher risk of mental ill health than heterosexuals, and these data support the need for policies that recognise coexisting health inequalities in LGB groups.^50^ Similarly, we found a strong association between reporting recent relationship breakup and treatment for depression, highlighting the need to consider relationship context in assessing patients’ mental health, and for proactive clinical management, which might include referral to counselling organisations like ‘Relate UK’. Depression was also more common in our study among another potentially vulnerable group, women reporting use of emergency contraception, which is an event that has been associated with depression in poor urban women, while the adverse outcome of unplanned pregnancy is a known threat to mental health.^51–55^ In the UK, women may readily obtain emergency contraception in pharmacies, where the capacity to assess mental health needs is likely to be limited by training and facilities to ensure confidentiality. Women accessing emergency contraception may, therefore, be in particular need of care in this respect, but research and policy are lacking in this area.

Our study is also relevant to sexual health practitioners; not only is the prevalence of mental health disorders high among patients attending sexual health clinics, but patients are more likely to reattend if staff recognise their psychological problems.^5^ Although the British Medical Association has called for greater importance to be given to mental health, and advocates for a holistic approach to care,^56^ the latest UK national guideline on consultations requiring sexual history-taking fails to emphasise mental health as an important comorbidity in patients requiring sexual healthcare.^57^ There are already pilot schemes, such as ‘Headspace’ in Australia, where mental and sexual health services have established collaborative partnerships to ensure full sexual health assessments, advice, treatment and referral as appropriate for clients.^58^ Our data support such integrated approaches to managing mental health and sexual health problems in primary care as well as specialist settings. Interventional studies might be required to understand whether such integrative approaches are a benefit to patients.

Overall, this study gives family doctors, who may be better placed to make links between services than other healthcare professionals, much needed information for understanding and improving the sexual health of patients treated for depression, and provides evidence to support the design of integrated approaches to managing patients with overlapping sexual and mental health needs.
