# Supplementary material for: Are depression and poor sexual health neglected comorbidities? Evidence from a population sample
Source: BMJ Open. 2016 Mar 23;6(3):e010521. doi: 10.1136/bmjopen-2015-010521 (PMC4809090; doi:10.1136/bmjopen-2015-010521)
Supplement: Supplementary appendix [file bmjopen-2015-010521supp.pdf]

Appendix 1: Sexual behaviour and STI risk of those reporting depressive symptoms, by gender

	**Men**		**Women**	
	Not treated and without depressive symptoms	Depressive symptoms (without treatment)	Not treated and without depressive symptoms	Depressive symptoms (without treatment)
Unweighted, weighted denominator	4809, 5981	405, 455	6413, 5718	549, 449
2+ heterosexual/same-sex occasions of sexual intercourse, past 4 weeks[1]				
%	68.7%	57.7%	66.1%	52.9%
OR (95% CI)	1.00	**0.62 (0.47 - 0.82)**	1.00	**0.58 (0.45 - 0.74)**
aAOR (95% CI)	1.00	**0.58 (0.44 - 0.77)**	1.00	**0.50 (0.39 - 0.64)**
2+ heterosexual partners, past year				
%	15.1%	21.6%	8.7%	13.0%
OR (95% CI)	1.00	**1.55 (1.18 - 2.05)**	1.00	**1.57 (1.22 - 2.01)**
aAOR (95% CI)	1.00	**1.45 (1.06 - 1.97)**	1.00	1.28 (0.99 - 1.67)
Same-sex partner, past 5 years				
%	2.5%	2.6%	2.8%	3.6%
OR (95% CI)	1.00	1.01 (0.52 - 1.94)	1.00	1.29 (0.82 - 2.04)
aAOR (95% CI)	1.00	0.97 (0.50 - 1.87)	1.00	1.13 (0.71 - 1.80)
2+ heterosexual/ same-sex partners without using a condom, past year				
%	7.4%	10.2%	4.4%	6.9%
OR (95% CI)	1.00	**1.41 (1.01 - 1.98)**	1.00	**1.63 (1.18 - 2.26)**
aAOR (95% CI)	1.00	1.29 (0.92 - 1.81)	1.00	1.32 (0.95 - 1.83)
Paid for heterosexual/ same-sex, past year				
%	1.1%	1.5%	0.1%	0.1%
OR (95% CI)	1.00	1.38 (0.59 - 3.24)		
aAOR (95% CI)	1.00	1.38 (0.58 - 3.25)		
Concurrent partnerships, past 5 years				
%	15.0%	20.7%	7.1%	8.8%
OR (95% CI)	1.00	**1.48 (1.11 - 1.97)**	1.00	**1.27 (0.92 - 1.74)**
aAOR (95% CI)	1.00	**1.39 (1.03 - 1.89)**	1.00	**1.05 (0.76 - 1.45)**
Know/ perceive their most recent partner had sex with somebody else, past 5 years[2]				
%	22.8%	29.3%	22.2%	32.5%
OR (95% CI)	1.00	**1.40 (1.06 - 1.86)**	1.00	**1.69 (1.30 - 2.18)**
aAOR (95% CI)	1.00	**1.37 (1.04 - 1.82)**	1.00	**1.65 (1.27 - 2.14)**
Diagnosed with a STI, past year				
%	0.9%	1.2%	0.8%	1.5%
OR (95% CI)	1.00	1.44 (0.64 - 3.23)	1.00	1.96 (0.88 - 4.35)
aAOR (95% CI)	1.00	1.21 (0.54 - 2.70)	1.00	1.37 (0.58 - 3.22)
Self-perceived risk of STI: Quite a lot or greater				
%	3.4%	7.4%	2.1%	4.4%
OR (95% CI)	1.00	**2.24 (1.52 - 3.31)**	1.00	**2.16 (1.36 - 3.42)**
aAOR (95% CI)	1.00	**2.09 (1.41 - 3.09)**	1.00	**1.91 (1.21 - 3.02)**

All participants who have had at least one opposite-sex or same-sex partner, ever (denominators may vary across variables due to item non-response)

aAOR: Age-adjusted odds ratio

[1] Denominator restricted to those reporting at least one heterosexual or same-sex partner in the last year (Men: Not treated and without depressive symptoms - 4809,5981; Depressive symptoms (without treatment) - 405, 455 | Women: Not treated and without depressive symptoms - 6413, 5718; Depressive symptoms (without treatment) - 549, 449)

[2] Denominator restricted to those reporting a sexual partnership in the past 5 years (Men: Not treated and without depressive symptoms - 3955, 3912; Depressive symptoms (without treatment) - 317, 256 | Women: Not treated and without depressive symptoms - 5273, 3657; Depressive symptoms (without treatment) - 438, 239)

Appendix 2: Sexual function of those reporting depressive symptoms, by gender

	**Men**		**Women**	
	Not treated and without depressive symptoms	Depressive symptoms (without treatment)	Not treated and without depressive symptoms	Depressive symptoms (without treatment)
Unweighted, weighted denominator	4750, 5914	396, 442	6334, 5650	540, 435
Lowest quintile of sexual function (ref)				
%	17.3%	41.1%	16.2%	38.1%
OR (95% CI)	1.00	**3.33 (2.51 - 4.41)**	1.00	**3.17 (2.45 - 4.10)**
aAOR (95% CI)	1.00	**3.63 (2.73 - 4.84)**	1.00	**3.49 (2.68 - 4.56)**
Components of the Natsal-SF score[1] (ref)				
Have experienced problems for 3+ months, past year				
%	39.3%	56.2%	46.7%	72.3%
OR (95% CI)	1.00	**1.99 (1.50 - 2.64)**	1.00	**2.98 (2.30 - 3.87)**
aAOR (95% CI)	1.00	**2.09 (1.57 - 2.78)**	1.00	**3.16 (2.43 - 4.12)**
Lacked interest in having sex for 3+ months, past year				
%	13.0%	27.8%	30.0%	52.8%
OR (95% CI)	1.00	**2.58 (1.88 - 3.54)**	1.00	**2.61 (2.05 - 3.33)**
aAOR (95% CI)	1.00	**2.63 (1.92 - 3.62)**	1.00	**2.77 (2.17 - 3.53)**
No orgasm or took a long time to reach orgasm despite arousal for3+ months, past year				
%	8.2%	11.2%	14.2%	24.9%
OR (95% CI)	1.00	1.42 (0.95 - 2.14)	1.00	**2.00 (1.52 - 2.64)**
aAOR (95% CI)	1.00	1.43 (0.95 - 2.15)	1.00	**1.96 (1.49 - 2.59)**
Trouble achieving/maintaining erections for 3+ months, past year				
%	12.2%	15.1%		
OR (95% CI)	1.00	1.29 (0.90 - 1.84)		
aAOR (95% CI)	1.00	**1.49 (1.03 - 2.17)**		
Disagree/ disagree strongly to the statement: "I feel satisfied with my sex life"				
%	15.1%	32.5%	11.2%	24.3%
OR (95% CI)	1.00	**2.71 (2.10 - 3.50)**	1.00	**2.53 (1.98 - 3.23)**
aAOR (95% CI)	1.00	**2.77 (2.14 - 3.58)**	1.00	**2.58 (2.01 - 3.30)**
Perceive a health condition/disability has affected sexual activity/enjoyment, past year				
%	13.5%	25.9%	12.8%	26.1%
OR (95% CI)	1.00	**2.24 (1.69 - 2.98)**	1.00	**2.39 (1.86 - 3.07)**
aAOR (95% CI)	1.00	**2.53 (1.89 - 3.40)**	1.00	**2.43 (1.89 - 3.12)**
Perceive medications taken have limited sexual activity/ enjoyment, past year				
%	6.2%	11.9%	3.8%	10.6%
OR (95% CI)	1.00	**2.04 (1.39 - 3.01)**	1.00	**2.99 (2.08 - 4.29)**
aAOR (95% CI)	1.00	**2.34 (1.55 - 3.52)**	1.00	**2.97 (2.07 - 4.27)**
Taken any medicine/ pills to assist sexual performance, past year				
%	6.2%	6.7%	0.7%	2.8%
OR (95% CI)	1.00	**1.10 (0.66 - 1.81)**	1.00	**4.22 (2.15 - 8.26)**
aAOR (95% CI)	1.00	**1.17 (0.70 - 1.95)**	1.00	**4.34 (2.21 - 8.51)**

All participants who have had at least one opposite-sex or same-sex partner, ever (denominators may vary across variables due to item non-response)

aAOR: Age-adjusted odds ratio

[1] All participants who have had at least one opposite-sex or same-sex partner, past year

Appendix 3: Sexual health service use of those reporting depressive symptoms, by gender

	Men		Women	
	Not treated and without depressive symptoms	Depressive symptoms (without treatment)	Not treated and without depressive symptoms	Depressive symptoms (without treatment)
Attended a sexual health (GUM) clinic, past year				
%	4.2%	6.2%	4.4%	8.3%
aAOR (95% CI)	1.00	1.23 (0.81 - 1.88)	1.00	**1.46 (1.04 - 2.06)**
AOR[1] (95% CI)	1.00	1.10 (0.71 - 1.72)	1.00	1.36 (0.94 - 1.97)
Had a chlamydia test, past year[2]				
%	15.6%	19.2%	25.1%	32.0%
aAOR (95% CI)	1.00	1.08 (0.74 - 1.57)	1.00	1.09 (0.84 - 1.42)
AOR[1] (95% CI)	1.00	1.00 (0.69 - 1.46)	1.00	1.04 (0.78 - 1.37)
Had a blood test for HIV, past year				
%	3.4%	5.9%	5.3%	6.9%
aAOR (95% CI)	1.00	**1.63 (1.00 - 2.68)**	1.00	1.06 (0.73 - 1.54)
AOR[1] (95% CI)	1.00	1.60 (0.96 - 2.67)	1.00	1.04 (0.72 - 1.52)
You or your partner used emergency contraception, past year				
%	2.3%	2.2%	1.5%	2.2%
aAOR (95% CI)	1.00	0.79 (0.44 - 1.40)	1.00	1.04 (0.60 - 1.78)
AOR[1] (95% CI)	1.00	0.75 (0.42 - 1.34)	1.00	0.85 (0.47 - 1.54)
Sought professional help regarding your sex life, past year				
%	6.4%	8.9%	5.7%	9.4%
aAOR (95% CI)	1.00	1.44 (0.93 - 2.23)	1.00	**1.70 (1.20 - 2.41)**
AOR[1] (95% CI)	1.00	1.51 (0.97 - 2.35)	1.00	**1.65 (1.16 - 2.34)**
Unweighted, weighted	4809, 5981	405, 455	6413, 5718	549, 449

All participants who have had at least one opposite-sex or same-sex partner, ever (denominators may vary across variables due to item non-response)

aAOR: Age-adjusted odds ratio

[1] Adjusted for age and 2+ partners without using a condom, past year

[2] Participants aged 16-44 years
